# What Research Evidence Is Valid for Psychotherapy Research?

**DOI:** 10.3389/fpsyt.2020.625380

**Published:** 2021-01-11

**Authors:** Björn Philips, Fredrik Falkenström

**Affiliations:** ^1^Department of Psychology, Stockholm University, Stockholm, Sweden; ^2^Department of Behavioral Sciences and Learning, Linköping University, Linköping, Sweden

**Keywords:** evidence, psychotherapy, evidence—based medicine, randomized controlled trial, process research, experimental methodology, causality, research design

## Abstract

Evidence-Based Medicine (EBM) have contributed to improved clinical practice with increased use of effective and life-saving treatments for severe diseases. However, the EBM model is less suitable for psychotherapy research than for pharmacological research and somatic medicine. The randomized controlled trial (RCT) design is an example of experimental methodology, which inevitably has more imperfections in psychotherapy research because psychotherapy RCTs cannot use double-blinding and the treatments tested are composite treatment packages. Long-term psychotherapy for severe and complex mental disorders is especially difficult to study with an RCT design. During the last decades, advanced analytic methods have been developed in psychotherapy process research, which enables investigation of causal connections regarding change mechanisms in psychotherapy. Therefore, we propose that the top of the research evidence hierarchy for psychotherapy should encompass: (1) RCT for circumscribed disorders, (2) cohort studies for complex disorders, and (3) advanced process studies for change mechanisms.

## Introduction

The evidence-based medicine (EBM) movement has had a major positive impact in many areas of medicine since it arose in the 1990's. The focus on critical appraisal of research findings as well as the development of systematic reviews and clinical practice guideline have contributed to improved practice of medicine with increased use of effective and life-saving treatments for diseases such as cancer and pneumonia (1). EBM introduced a hierarchy for appraisal of research evidence, with the randomized controlled trial at the top with regard to original studies (2). The EBM model has been developed over time, for example with the Grades of Recommendation Assessment, Development, and Evaluation (GRADE) system, in which factors such as study quality and effect size are included in the appraisal (3). However, we claim that the EBM model is less suitable for psychotherapy research than for pharmacological research or other areas of somatic medicine. With this article, we will argue that some modifications of EBM are needed in the area of psychotherapy for mental disorders. Several suggestions have been proposed for modifications of EBM for psychotherapy (4–6). However, we believe that these have not gone far enough in revising the hierarchy of evidence for psychotherapy for mental disorders.

## The Randomized Controlled Trial

As above, the randomized controlled trial (RCT) is considered the gold standard for testing the efficacy of a treatment for a particular disorder and thus the foundation for establishing whether that treatment is evidence-based (1, 7). Many influential scholars promote RCT design as the gold standard also for psychotherapy research, based on the argument that RCT is the only research design that can establish causality by ruling out alternative explanations (8–10). The logic behind the RCT design is to maximize internal validity, i.e., to use an experimental methodology in which alternative explanations are eliminated in order to establish a causal connection between the independent variable (treatment) and dependent variable (outcome). In clinical trials testing a pharmacological treatment, placebo control and double-blinding are used in order to rule out expectancy effects as alternative explanations of outcome. The patient, the physician, and the nurse are unaware of which patients receive the actual medication and which patients receive placebo pills (11). However, such double-blinding is practically impossible in psychotherapy research (4). Sometimes it is possible to keep the patient unaware of what specific psychotherapy he/she is receiving, but this is increasingly difficult as patients with mental disorders have become more savvy about different psychotherapy methods (which sometimes leads to patients having a strong preference for a particular type of therapy). Furthermore, it is impossible to keep a psychotherapist unaware of which type of psychotherapy he/she is practicing. Hence, expectancy effects are impossible to eliminate in psychotherapy research, which implies that the RCT design is not as suitable for psychotherapy as it is for pharmacological treatment.

To achieve the aims of maximizing internal validity in a psychotherapy RCT, the trialist needs to ensure that (1) the sample is homogenous and clearly specified (usually in terms of psychiatric diagnosis), (2) the treatment method is clearly specified in a treatment manual, (3) therapists are adequately trained in the treatment method(s) that is being tested, and (4) therapists perform the treatment adequately (using audio or video-based adherence ratings) (8). However, many authors have pointed out how procedures designed to maximize internal validity tend to lead to poor external validity, i.e., to poor generalizability of the findings to settings outside the experimental situation (12). Lambert described how naturalistic outcome studies carried out in an ordinary clinical setting (effectiveness studies) have stronger ecological validity by using alternative procedures, for example: (a) including regular patients at the clinic with less strict eligibility criteria; (b) being more flexible regarding length and “dose” of therapy in order to adapt to the particular patient; (c) allowing more freedom to the therapist in how to apply the psychotherapy method; and (d) including therapists who work at the clinic who are not hand-picked for the research study (13). Efforts have been made to increase the external validity in RCTs by introducing the concept “pragmatic clinical trials,” in which broad eligibility criteria are used in order to include real-world patients and clinical procedures are consistent with usual clinical care. External validity is indeed stronger in pragmatic clinical trials but as Ware and Hamel note: “they sacrifice internal validity to achieve generalizability” (14).

An additional problem is that RCTs most often test the efficacy of an entire treatment package. Psychological treatments such as cognitive behavioral therapy (CBT) or psychodynamic therapy (PDT) are very dissimilar to psychotropic medications such as fluoxetine or olanzapine, with regard to the psychological treatments being based on a number of central therapeutic principles being actualized through a conglomerate of different therapeutic interventions that the therapist uses in the sessions together with the patient. These specific therapeutic principles and interventions are interwoven with more generic efforts to establish and maintain a helpful therapeutic relationship, through the therapist's attitude of empathy, validation, cooperation, et cetera (15). Hence, a randomized controlled trial involving a psychological treatment (e.g., CBT vs. waiting list) might produce the result that the treatment package is efficacious, but we still do not know what made the treatment work. What were the crucial therapeutic interventions and change mechanisms that helped the patients to improve? Kazdin refers to this as a lack of construct validity in RCTs of treatment packages (16). Thus, RCTs are more appropriate for single-component treatments for patients with one circumscribed problem than for composite, interactive (sometimes long-term) psychotherapies for patients with severe and/or comorbid mental disorders. However, single-component psychotherapy treatments are rare, and even the ones that exist (e.g., exposure treatment for phobias) probably rely at least to some extent on common factors such as the establishment of a working alliance and therapist empathy (17). Moreover, the number of patients with a single, circumscribed problem is probably very small—at least in specialist psychiatric departments.

Another important point about psychotherapy RCTs is that experimental group designs do not establish absolute natural laws of psychology. Instead, they establish probabilistic laws in terms of the average causal effect for the average patient (18). Here we have a problem of generalizability: a clinician who is about to choose a treatment for a new patient might not know how close or how far from the average this particular patient is (19). Some authors claim that promoting RCT as the gold standard for evidence has not advanced progress toward more effective psychotherapy, instead evidence suggests decreased pre-post effect sizes over time and a greater divide between research and practice (20).

Another major critique of experimental designs in psychotherapy research is that they might be based on incorrect epistemological assumptions. Both experimental case studies and RCTs are based on the assumption of linear causality, i.e., experimental studies test hypotheses of the type “A causes B” (equivalent to the scientific laws of Newtonian mechanics). However, it is highly likely that the human mind and the practice of psychotherapy would better be characterized as complex systems (equivalent to quantum physics, meteorology, or economics), which indicates that psychotherapy research should rather use models like non-linear dynamics and chaos theory. To complicate things further, the human mind contains elements that are not present in even the most complex natural sciences—elements that are likely to influence the process and outcome of psychotherapy, such as consciousness, intentionality, subjectivity, and agency. As human beings possess agency and as psychotherapies are collaborative work between patient and therapist characterized by responsiveness toward the other and thus bi-directional effects, it is a misconception to label psychotherapy as independent variable and patient symptoms as dependent variable (19). To analyze such complex interplay of multiple factors we need the type of advanced psychotherapy process research that we will describe later. Recently, researchers leaning on complexity theory have used computational models to, for example, formalize ideographic theories of functional analysis for panic disorder and to test a perceptual control theory account of psychological change (21, 22).

## Particular Research Challenges for Psychotherapy for Complex Psychopathology

Many psychotherapy researchers have experienced that the RCT design is difficult to use for patients with more complex psychopathology (19). As an illustrative example, let us consider patients with concurrent borderline personality disorder (BPD) and substance use disorder (SUD), which was the target group for a pragmatic RCT conducted by one the authors. Patients with such severe psychopathology are often traumatized, and lacking trust and hope that treatment providers can help them. It often takes a long time to build a secure relationship with such individuals and to strengthen their motivation to dare begin psychotherapy and work with their problems. At that moment, randomization is detrimental to the patients who end up in the control condition and do not receive active treatment. Our study of mentalization-based treatment (MBT) vs. standard SUD treatment was aiming for the inclusion of 80 patients (based on a priori statistical power calculation), but after 5 years of hard work and consuming all of the external research funding, we had to stop at *N* = 46 (23). In fact, other RCTs of psychotherapy for concurrent BPD and SUD had even smaller sample sizes (24–27).

In our view, the just-mentioned small sample sizes are an indication that psychotherapy with fragile patients should be studied with research designs other than a typical RCT. Our example was dual diagnosis, but the same argument would be valid for disorders such as severe personality disorders (e.g., paranoid, schizoid, schizotypal, or narcissistic), complex PTSD and dissociative disorders, as well as comorbidity of severe disorders. A variant of RCT that might be appropriate is the one comparing two bona fide psychotherapy methods so that none of the patients received inactive therapy. But such a comparative RCT would be extremely expensive if it were to meet design needs for sufficient statistical power and adequate treatment duration (probably more than 1 year). Also, when there is no existing treatment with established empirical support for a particular pathology, the first step of gathering evidence for a novel treatment would be to test it against a control condition (such as a placebo treatment). A more productive strategy might be to declare another gold standard research design for patients with complex psychopathology, for example cohort studies with repeated measurement of outcome and process variables and comparison to a benchmark (28). As the clinical reality is complicated with no sharp line between circumscribed and complex psychopathology, as well as varying associations between comorbidity and severity (or functional impairment), such new standards might have to be based on continuums of complexity/severity/impairment rather than distinct categories. Further complications are the large heterogeneity within diagnostic categories and growing evidence that a general psychopathology factor might contribute to all mental disorders (29).

In our study of MBT vs. standard SUD treatment for dual diagnosis patients, we found no significant effect of MBT, partly because the staff taking care of the control group helped many of those patients by referring them to some sort of psychotherapy (23). That was good news for the patients, but it was bad news for our research study and for the RCT design!

## Causal Inference from Psychotherapy Process Research

Since RCT designs leave a knowledge gap about the change mechanisms in psychotherapy, this gap has to be filled by research focusing directly on this question. Partly this can be done by using the experimental component designs with dismantling or constructive strategies (16). A problem with these designs, however, is that since the effect size of a single therapy component is likely to be fairly small, and the effect of so-called unspecific effects/common factors (including but not limited to placebo) is likely to be large, they require huge sample sizes (17, 30).

Haynes and O'Brien indicate the following four requirements for causal inference:

1. The variables must covary.
2. The hypothesized causal variable must precede the outcome variable.
3. Realistic alternative explanations for the observed covariance must be reasonably excluded.
4. There must be a plausible explanation for the hypothesized causal relation (31).

A well-conducted RCT meets all of these requirements, which is the reason it is considered the gold standard for causal inference. We would argue that psychotherapy process research is increasingly able to meet most of these requirements, and methodological developments to meet them all are well under way. Since such research can be done on naturalistic data as well as on RCT data, it is less subject to the limitations of RCTs that we have outlined above. Process research also has the advantage of giving much more specific information to the therapist about what to do during the therapy sessions compared to a difference between two group means regarding two treatments or comparing one treatment against a control condition. A common misunderstanding is that process research is only meaningful after having established efficacy for a treatment package using RCT. If this was true, the usefulness of process research would be much more limited than we propose. However, even if a treatment package has unknown efficacy, or even if has been shown to be inefficacious overall, process research can still be used to identify the ingredients that are more/less efficacious.

In the last decade, panel data/cohort designs with repeated measurements of mechanisms and outcome across therapy for a number of patients, have become state-of-the-art for psychotherapy process research (32, 33). These designs meet most of the requirements for causal inference mentioned above and could be labeled “mechanisms of change research.” Covariation (1) is studied in all quantitative research, and the requirement for plausible theoretical explanations (4) is also not difficult to meet. Temporality (2) has traditionally been problematic in process designs in which a process was simply related to pre-post outcome, but with session-by-session measurements of process and outcome time-lagged associations can be analyzed, ensuring that this requirement is met (34). The most difficult requirement for causal inference is ruling out alternative explanations (3), or the risk for third-variable confounding. Most researchers are aware that potential confounders can be measured and included as covariates in multivariate statistical models, but what to do about confounders that are not measured, perhaps even not known? This is where the power of randomization comes in, but there are statistical ways of achieving this when randomization is not feasible. Modern cross-lagged panel models enable the separation of average differences in processes and outcomes between patients from over-time fluctuations within patients, which ensures that confounders that are stable over time—even ones that are unobserved—can be ruled out (35). This means that these designs are approaching the RCT in terms of the potential for causal inference, while not being marked by the disadvantages of these.

Although these designs protect against confounders that are stable over time, there are a number of potential confounders that vary over time, e.g., certain therapist techniques, relationship variables, patient motivation etc. Recent developments in statistical methods in other scientific fields (most prominently in economics, sociology, and biology) may, under certain assumptions, enable even such confounding to be ruled out. For instance, instrumental variable regression has been used for causal inference in econometrics for a long time, probably since the 1920's (36). This method requires fairly strong assumptions, and it is difficult in practice to find instruments that satisfy these. However, recent developments enable researchers to relax those assumptions, and research is under way testing the performance of these methods (37).

An example of such process research took place in analyzing data from the above-mentioned trial on MBT for comorbid BPD and SUD. In the study, we used a microanalytic sequential process design, showing that within a session, therapist interventions directed at exploring mental processes were connected with a subsequent higher patient level of mentalization. In other words, therapist interventions guiding the patient to explore mental processes lead to increased patient mentalizing, supporting this theoretically proposed change mechanism in MBT (38). In this study, covariation [criterion (1)] is estimated using regression models of patient mentalizing on therapist interventions, correct temporality [criterion (2)] is ensured by using therapist interventions that immediately precede patient mentalizing statements, alternative explanations [criterion (3)] are partly ruled out by the design and statistical model, e.g., by separating within-person variation in mentalizing/interventions from stable between-person differences so that the only possible confounders left are ones that co-vary with therapist statements and fluctuations in patient moment-to-moment mentalization. Finally, the study hypotheses are based on mentalization theory [criterion 4)].

Such rigorous process research can be based on psychotherapy sessions from RCTs or naturalistic studies. An advantage of using data from RCTs is that they have more well-defined patient samples and treatment methods; hence, it is easier to know which generalizations of the findings are adequate. However, as mentioned, less strict designs often have the advantage of increased ecological validity.

## Discussion

As we have shown, the RCT is the strongest research methodology for testing treatment efficacy (9, 10). However, the more complex the treatments and the patients' psychopathologies are, the more difficult to use the RCT design gets. As psychotherapy methods almost invariably are complex treatment packages, the RCT design also misses the critical question of interest, namely: what specific treatment principles and interventions are helpful for the patient, or in other words what are the crucial change mechanisms? Based on these arguments we suggest some modifications of the EBM model concerning psychotherapy for mental disorders. Our proposal is close to Castonguay and Beutler's concept “principles of therapeutic change that work” and Salkovski's concept “empirically grounded clinical interventions” (39, 40).

We propose that the following three designs should have an equal place at the top of the hierarchy for research evidence concerning psychotherapy:

1. For short-term psychotherapy for patients with circumscribed, less severe psychiatric disorders, RCT is still the research design of choice.
2. For long-term psychotherapy for patients with severe or complex psychopathology, cohort study with repeated measurements and comparison to a benchmark is the research design of choice.
3. In order to investigate the crucial therapeutic principles and change mechanisms in psychotherapy for particular mental disorders, process research using stringent strategies to establish causal connections and appropriate statistical analytic methods is the research design of choice.

## Author Contributions

BP wrote most of the introduction, the part about RCTs, and the discussion. FF wrote most of the part about process research. We have sent the developing manuscript back and forth to each other and made revisions. The submitted version is approved by both. Both authors have contributed to the manuscript.

## Conflict of Interest

The authors declare that the research was conducted in the absence of any commercial or financial relationships that could be construed as a potential conflict of interest.
